# Impact of cost distance and habitat fragmentation on the daily path length of *Rhinopithecus bieti*

**DOI:** 10.7717/peerj.9165

**Published:** 2020-05-20

**Authors:** Cong Li, Xumao Zhao, Dayong Li, Paul Alan Garber, Zuofu Xiang, Ming Li, Huijuan Pan

**Affiliations:** 1 School of Ecology and Nature Conservation, Beijing Forestry University, Beijing, China; 2 CAS Key Laboratory of Animal Ecology and Conservation Biology, Institute of Zoology, Beijing, China; 3 Institute of Innovation Ecology, Lanzhou University, Lanzhou, China; 4 Key Laboratory of Southwest China Wildlife Resources Conservation [Ministry of Education] China West Normal University, Nanchong, China; 5 Department of Anthropology and Program in Ecology and Evolutionary Biology, University of Illinois, Urbana, IIIinois, USA; 6 College of Life Science and Technology, Central South University of Forestry and Technology, Changsha, China; 7 Center for Excellence in Animal Evolution and Genetics, Chinese Academy of Sciences, Kunming, China

**Keywords:** *Rhinopithecus bieti*, Cost-distance model, Habitats fragmentation, Daily path length, Seasonal variation of habitat, Ranging behavior, Landscape heterogeneity, Primates, Human disturbance, Spatial analyst

## Abstract

An understanding of primate movement patterns in response to natural and anthropogenically induced changes in habitat heterogeneity, food availability, and plant species distribution is essential for developing effective management and conservation programs. Therefore, from July 2013 to June 2014, we examined the effects of landscape configuration on the ranging behavior (daily path length, DPL) of the Endangered Yunnan snub-nosed monkey (*Rhinopithecus bieti*) in the Baimaxueshan National Nature Reserve (27°34′N, 99°17′E) in Gehuaqing, China. Given the extreme difficulties in following the study group across high altitude mountainous terrain across an elevation of 2,500–4,000 m, we were only able to collect DPL using 3-4 GPS points per day on 21 individual days. We found that *R. bieti* traveled the shortest DPL in winter (1,141.31 m), followed by spring (2,034.06 m) and autumn (2,131.19 m). The cost distance, a statistical tool designed to estimate the difficulty of a species moving across its distributional range, was lowest in autumn (205.47), followed by spring (225.93) and winter (432.59) (one-way ANOVA: *F* = 3.852, *P* = 0.026, df = 2). The habitat fragmentation index (HFI), which measures the density of forest patches, indicated areas visited in the winter were more fragmented (HFI = 2.16) compared to spring (HFI = 1.83) or autumn (HFI = 1.3). Although our results should be considered preliminary, they suggest that both the availability of suitable travel routes and habitat fragmentation, driven by high-intensity human disturbance, constrain the movement of *R. bieti*. We found that undisturbed areas of the bands’ range contained a high density of lichens, which represent a nutritious and abundant and year-round food source for Yunnan snub-nosed monkeys. In order to protect this Endangered species, we recommend that researchers construct detailed maps of landscape heterogeneity, particularly habitat connectivity, forest fragmentation, and seasonal variation in the location of major food patches in order to better understand and mitigate the effects of seasonal habitat change on patterns of *R. bieti* habitat utilization and population viability.

## Introduction

Multiple studies have shown that increasing habitat loss and fragmentation pose continuing threats and challenges to biodiversity conservation (Martínez-Ramos et al., 2016; Wu, 2013). Fragmented landscapes are described as discontinuous patches of original or impacted habitats that, depending on their size, location, edge characteristics, proximity to human settlements and the movement and dispersal requirements of a given animal or plant species, can result in isolated or island habitats and reduced population viability (Newman et al., 2013; Simpson & Weiner, 1989). Factors contributing to habitat fragmentation include land reclamation, deforestation, cultivation (Faleiro, Machado & Loyola, 2013; Tang et al., 2013; Zhang, Lu & Guan, 2012), the creation of pastures, and along with the added effects of climate change (Asner, Loarie & Heyder, 2010; Mantyka-Pringle, Martin & Rhodes, 2013), reduce biodiversity and increase the risk of animal and plant extinctions (Estrada, Raboy & Oliveira, 2012; Haddad et al., 2015; Marsh et al., 2013). In addition, large-scale habitat fragmentation is a landscape-level spatial process that can divide what was previously a geographically widespread species into genetically isolated subpopulations (Fahrig, 2003). With increased fragmentation, the spatial heterogeneity of the landscape becomes more complex and the resistance of different landscapes to the movement of species changes, which affects patterns of migration, dispersal, and gene flow (Laurance et al., 2002; Saunders, Hobbs & Margules, 1991; Zhang & Ma, 2014). Recent studies have tried to characterize the ability of species to move between different patches across the landscape by using cost distance modelling (Howard et al., 2015; Li et al., 2017). Cost distance modelling is a statistical tool designed to estimate the difficulty of a species moving across its distributional range by assigning resistance values for each pixel of a rasterized map and, using a species perspective approach, take into account landscape configuration and physical structure to evaluate landscape connectivity and its effects on the energetics of animal movements (Richard & Armstrong, 2010). Although the model does not measure energetic costs directly, it can assess whether the chosen steps differ from a random sample of alternatives of similar length (Richard & Armstrong, 2010).

In the case of nonhuman primates, recent human activities including hunting and the bush meat trade, industrial agriculture, deforestation, mining, cattle grazing, dam building, and the construction of road networks in previously inaccessible areas have resulted in a significant loss of suitable habitat and increasing spatial conflict with local human populations. A recent assessment found that approximately 60% of primate species are threatened with extinction and 75% have declining populations (Estrada et al., 2017). And, although some primate species may be able to survive temporarily in highly fragmented habitats (Mekonnen et al., 2017), the ability of a given primate species to adjust to habitat fragmentation is dependent on factors such as dietary and digestive requirements, fragment size and connectivity, group size and cohesion, the likelihood of individuals traveling on the ground while crossing large open spaces, and the size, distribution, and availability of suitable feeding sites. In this regard, different species adopt different foraging and dietary strategies to cope with forest fragmentation and seasonal changes in food distribution and availability. For example, groups of Geoffroyi’s spider monkeys (*Ateles geoffroyi*) living in fragmented forests devoted more time to foraging (48% of activity budget vs. 40% of activity budget), compared to groups of the same species living in continuous forest (Chaves, Stoner & Arroyo-Rodríguez, 2011). Also, Milne-Edwards sifaka (*Propithecus edwardsi*) traveled longer distances per day in fragmented forest (818 m in fragmented forest vs. 747 m in continuous forest) than they did in continuous forest (Gerber et al., 2012). In contrast, groups of black bearded sakis (*Chiropotes satanas*) (Boyle & Smith, 2010) adopted a different strategy and devoted more time to resting in fragmented forests (60% activity budget in fragmented forest and 40% of activity budget in intact forest). In the case of the Diademed sifaka (*Propithecus diadema*), group members traveled shorter distances per day (837 m vs. 987 m) in fragmented habitats than did individuals living in intact forests (Irwin, 2008). Thus, different primate species respond differently to the challenges of exploiting fragmented habitats. In addition, although some primates such as bonobos (*Pan paniscus*) avoid areas of human disturbance and fragmentation (Hickey et al., 2013), other species are able, at least in the short-term, to adjust their behavior and persist in fragmented and marginally degraded habitats. For example, Mekonnen et al. (2017) found that Bale Mountain vervet monkeys (*Chlorocebus djamdjamensis*) were able to successfully inhabit fragmented forests surrounded by a matrix composed of agricultural fields, pastures, and human settlements by adopting a behavioral pattern that included both crop raiding and consuming common plant foods in these altered habitats.

An understanding of temporal variation in animal movement and habitat utilization, in response to factors such as seasonal changes in food availability (Wartmann, Purves & Schaik, 2010), temperature (Ren et al., 2009b) and rainfall (Scholz & Kappeler, 2004) offer insight into primate behavioral flexibility and the manner in which individuals’ of a given species adjust their behavior to successfully exploit changing environmental conditions (Garber et al., 2019). For example, in the case of the black and gold howler monkey (*Alouatta caraya*), during summer when ripe fruits were available the howler’s daily path length (DPL) increased by approximately 200 m (26%) compared to the DPL in spring when mature leaves were the primary dietary component (Raño et al., 2016). This minor adjustment in howler ranging behavior enabled individuals to locate and exploit a more widely scattered and dispersed resource. Thus, examining changes in animal movement patterns in response to temporal changes in resource availability and distribution in both intact and fragmented landscapes is critical for proposing effective conservation solutions, including programs of reforestation and the establishment of dispersal corridors (Estrada, 2006).

The Yunnan snub-nosed monkey (*Rhinopithecus bieti*, current population estimate is <3,000 individuals remaining in the wild) is an Endangered species of Asian colobine or leaf-eating primate (all colobines exhibit a large sacculated stomach with a PH of 5-7, and a microbiome specialized for the digestion of cellulose and hemicellulose (Lambert, 1998)) endemic to southwestern China (IUCN, 2011; Kirkpatrick, 1995). They inhabit the highest elevation of any non-human primate, ranging up to 4,500 m (Long et al., 1994). Their distribution range includes alpine mixed broad-leaved coniferous forests and pure coniferous forests (Grueter et al., 2013). Like many primates, the habitat of Yunnan snub-nosed monkey is in the process of severe degradation and fragmentation (the suitable habitat area available to them is expected to decrease by 8.0%–22.4% by the year 2050) due to increased human activities, such the collection of forest products and the conversion of natural forest to pasture land for cattle grazing (Zhao et al., 2019). Several previous studies have focused on the effects of food availability and distribution on the movement patterns of Yunnan snub-nosed monkey. For example, in the winter when many trees lose their leaves *R. bieti* was found to increase foraging time from approximately 30% of their activity budget in summer to 47% in winter (Grueter et al., 2013), and to decrease resting time from approximately 37% of their activity budget in summer to 20% in winter (Grueter et al., 2013). In contrast, daily path length (DPL) during the winter was 985 m, which is the shortest among all seasons of the year, suggesting that winter resources exploited by Yunnan snub-nosed monkey are distributed in large and concentrated food patches (Grueter et al., 2013). Moreover, the Yunnan snub-nosed monkeys exploit extremely large home ranges. However, the home range area used during the fall (9.3 km^2^) was significantly smaller than the home range area exploited during other seasons (spring: 17.8 km^2^; summer: 18.6 km^2^; winter: 18.2 km^2^) (Grueter et al., 2008). Thus, it appears that during most of the year, the Yunnan snub-nosed monkey exhibits a pattern of habitat exploitation in which a relatively small core area is used intensively over periods of days or weeks, and then individuals travel to a new or distant part of their range and exploit that area intensively.

The goal of our study is to expand the currently available knowledge of the Yunnan snub-nosed monkey by examining the relationship between fragmented landscape, habitat utilization, and ranging behavior. Specifically, we calculated the DPL of Yunnan snub-nosed monkeys and used cost distance modeling and the landscape index to evaluate the effects of habitat fragmentation on seasonal differences in ranging behavior. An understanding of how the Yunnan snub-nosed monkey an Endangered primate species, adjusts its behavior in response to seasonal changes in resource availability and distribution, and differentially exploits intact and anthropogenically disturbed habitats is critical for developing effective conservation and management policies to protect this species and its environment.

## Methods

### Ethical standards

All research methods adhered to Chinese legal requirements and complied with protocols approved by the State Forestry Administration of China and the American Society of Primatologists principles for the ethical treatment of primates.

### Study site and data collection

**Figure 1:**
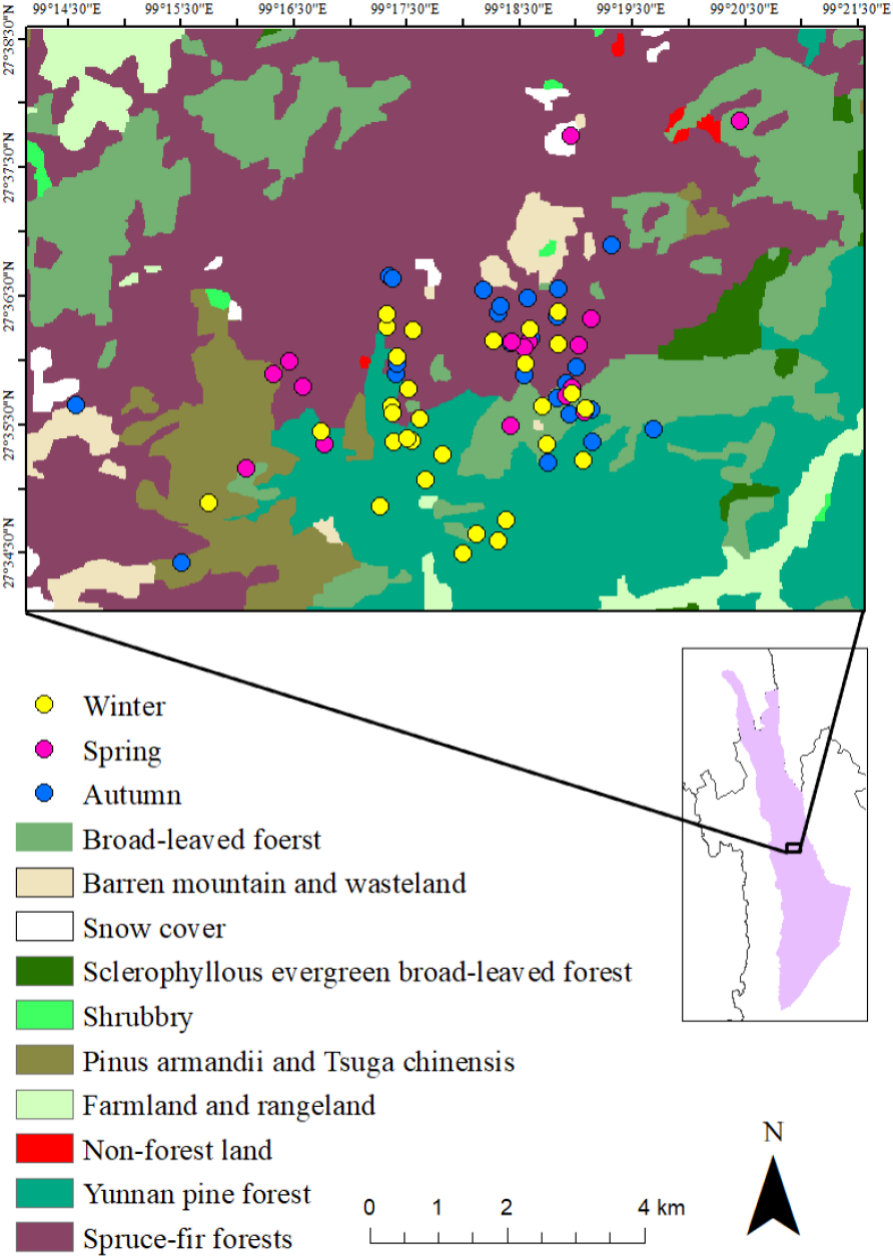
Study area, vegetation distribution and the activity points of *R. bieti* in different seasons.

The research site is the Baimaxueshan National Nature Reserve, in Weixi, Tacheng, Gehuaqing (27°34′N, 99°17′E), Yunnan Province, China (Fig. 1). The elevation of the study area is between 2,600-4,000 m.

The vegetation in the research area can be divided into six categories: (i) Yunnan pine (*Pinus yunanensis*) forest (2,500–3,200 m); (ii) Evergreen broad-leaved forest (2,500–3,000 m), (iii) Montane sclerophyllous oak forest (3,200–3,500 m); (iv) Coniferous and broad-leaved mixed forest (Armand pine, *Pinus armandii* and Chinese hemlock, *Tsuga chinensis*, 2,900–3,600 m); (v) Dark conifer forest (Spruce-fir forests, 3,500–4,000 m); (vi) Non-forest vegetation, farmland, and barren land. Among the six vegetation types, only two, Coniferous and broad-leaved mixed forest and Dark conifer forest are suitable habitats for the Yunnan snub-nosed monkey (Kirkpatrick et al., 1998; Li et al., 2008; Xiao et al., 2003). Ren et al. (2009b) adopted Zhang’s classification (i.e., daily mean temperatures (*T*) ≥ 22 °C was scored as summer, *T* ≤ 10 °C was scored as winter and 10 °C <*T* < 22 °C spring and autumn) to recognize three seasons in this area: spring (May to July), autumn (August to October) and winter (November to April). These seasons differ in rainfall and temperature. Highest temperatures and greatest rainfall occur in July and August (spring and autumn), whereas the lowest temperatures and rainfall occur in December and January (winter) (Li et al., 2013).

Our *R. bieti* breeding band, consisted of approximately 407 individuals, and is habituated to human observers. The social organization of *R. bieti* is best described as a multi-level society (MLS), which is principally composed of from 14–18 one-male units (OMUs) and at least one all-male unit, which are collectively referred to as a breeding band. OMUs contain one adult male, from three to five adult females and several immature offspring. Males emigrate the OMU as juveniles (around age 4) and enter an all-male unit (AMU) which is composed of juvenile, subadult, and adult bachelor males. The number of bachelor males in the AMU ranged from 10–20. Typically, when the band travels, it is followed by one or more AMUs across its range. From July 2013 to June 2014, we recorded the location of the geographic center of the breeding band using a handheld Garmin GPS Map 62 s Geographic Positioning System (GPS). We followed the monkeys from 6:00 until they entered their sleeping tree on 21 days. Day length varied from 10 h in December to 14 h in June. In December, the monkeys entered their sleeping tree at 16:00 and in June the monkeys entered their sleeping site at 20:00. In order to determine the geographic center of the band, we were positioned 30 to 50 m away from the center of the band and then moved to the center location to record the GPS point. Ninety-three percent of our GPS data points were recorded in this way. On 7% of occasions, because of inclement weather and difficult-to-navigate terrain characterized by steep mountain slopes, we collected the GPS point at a distance of more than 100 m from the center of the band. Each GPS record included the location, altitude, and vegetation type based on the six habitat categories listed above. On average we recorded 3–4 GPS points per observation day. Data collection was accomplished with the assistance of local guides and reserve staff. Our field assistants had extensive experience in field tracking monkeys and in data collection, however, we did not test for interobserver reliability. We acknowledge that in recording only 3-4 GPS points per day and in obtaining data for only 21 complete days, the results presented in our study are best considered preliminary.

### Data analysis

To calculate the DPL of the group, we converted GPS points to Shape files in ArcGIS 10.3 (Esri, Redlands, CA, USA) (https://www.arcgis.com). We screened the data to remove erroneous points (those that were clearly outside our study area). Our sample included 70 GPS points of observational data. This included 16 points in spring, 25 points in autumn, and 29 points in winter. The land-use map of the study area was added into the point Shape file via ArcGIS.The DPL during different seasons was calculated using the spatial analysis feature in ArcGIS 10.3. During spring we calculated DPL on five days (May: one day; June: two days; July: two days), in autumn on seven days (August: three days; September: two days; October: two days) and in winter during nine days (November: three days; March: three days; April: three days). We use the measurement tools to calculate the average DPL per season.

Cost distance was calculated using the “cost-distance” tool in ArcGIS 10.3, which simulates the accumulative cost distance for each cell based on its resistance surface score, as the species travels across its range. The resistance surface is also known as the cost-raster. Each cell, which measures 20 × 20 m, of the cost-raster is given a “cost” based on the habitat present in the cell. The cost of land use is defined as the resistance of different landscape types to the movement of the species (Perović et al., 2010). In this study, based on the research of Wu et al. (2009) and Chardon, Adriaensen & Matthysen (2003), we set resistance surface cost to two fixed values, giving the most suitable landscape types and the most unsuitable landscape types a cost value of 1 and 100, respectively. In addition, based on the results of Li et al. (2014), we assigned a cost of 1 to optimal habitat for *R. bieti* (Spruce-fir forests, Coniferous and broad-leaved mixed forest), a cost of 50 (shrubbery, Sclerophyllous evergreen broad-leaved forests) and 60 (Broad-leaved forests) to suitable habitats, a cost of 70 to unsuitable habitats (barren land, Non-forest land and Yunnan pine forests), and a cost of 100 to habitats containing barriers to movement (farmland and rangeland). The land-use map (20 × 20 mresolution) we used is derived from an assemblage of 13 SPOT 5 satellite images (November 2004, 60 × 60 km) covering the study area. We verified our habitat classification accuracy using data from the Conservation Information Centre of The Nature Conservancy of China. We generated the cost distance distribution map with the GPS points in different seasons using ArcGIS 10.3. We assumed that if the band was located in a particular habitat type when a GPS point was taken, that the band remained in that habitat type until the next GPS point was taken. On average, successive GPS points were taken four to six hours apart. One-way ANOVA tests were performed using SPSS 25.0 (IBM, Armonk, NY, USA).

Fragmentation analysis was conducted using the software FRAGSTATS 4.2 (McGarigal, Sam & Ene, 2012). We defined patch density (PD) using the fragmentation index. The fragmentation index is defined as the number of patches (a relatively homogeneous area that differs from its surroundings) per unit area (standardized to 100 ha in FRAGSTATS). The patch density is given by:

(1) \begin{eqnarray*}& & \mathrm{PD}=\mathrm{N/ A},\end{eqnarray*}

where *N* is the number of patches and A is the total area of habitats. PD varies from 0 to infinity. Larger PD values indicate that the habitat is more fragmented but does not take into account the size of each patch (McGarigal & Marks, 1995; Saura & Martinez-Millan, 2001).

## Results

The breeding band primarily exploited spruce-fir forest in spring (50%) and autumn (64%), followed by broad-leaved forests (24% and 28.75% respectively). In winter, Yunnan pine forest was used as primary habitat (55.67%), followed by spruce-fir forest (26.67%) (Fig. 1). Given that Spruce-fir forest accounted for 45.07% the group’s home range, Yunnan pine forest, 29.58%, broad-leaved forests, 18.31%, and *Pinus armandii* and *Tsuga chinensis* forest, 5.63%, the results indicate that the Yunnan snub-nosed monkeys inhabited different areas of their range (based on elevation and forest type) during different times of the year, with spring and autumn being the most similar and winter being the most different.

**Figure 2:**
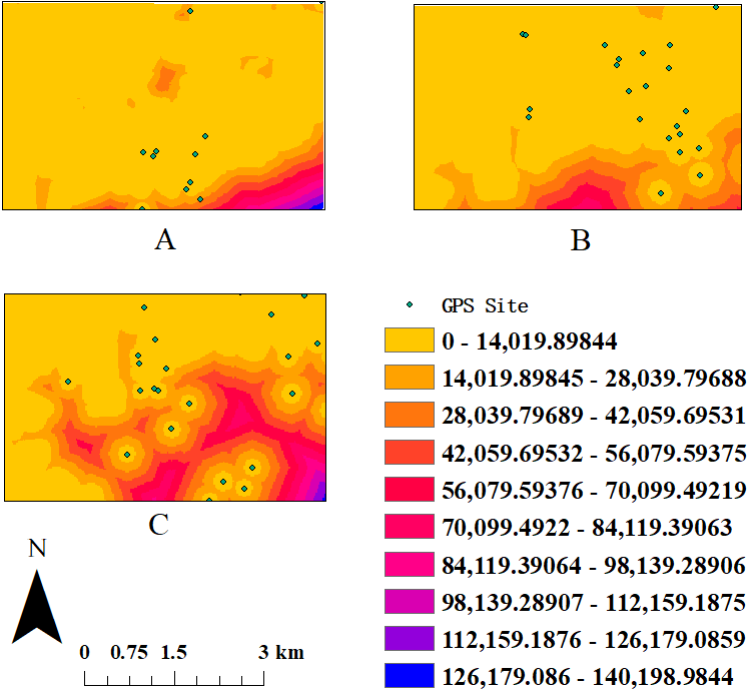
The sketch map of cost distance-grid in different seasons. (A) The activity area in spring. (B) The activity area in autumn. (C) The activity area in winter.

Seasonal mean DPLs and cost distance both are listed in Table 1. Cost distance varied significantly across seasons (one-way ANOVA: *F* = 3.852, *P* = 0.026, *df* = 2), being greater in winter than during spring or fall (Fig. 2). Seasonal DPLs showed no significant differences (one-way ANOVA: *F* = 1.652, *P* > 0.1, *df* = 2) but the shortest mean DPL occurred during winter. The habitat fragmentation index (PD) was highest in winter (2.16), followed by spring (1.83) and autumn (1.3) (Table 1), suggesting that the distribution of resources was most fragmented during the coldest period of the year.

**Table 1:** Results of DPL, cost distance, and selected landscape indicators.

Seasons	*n*	PD (N/A)	DPL (mean ± sd, m)	Cost distance^*^	
				Mean	Range
Spring	16	1.83	2,034.06 ± 1,638.53	225.93	4∼1,457.02
Autumn	25	1.30	2,131.19 ± 1,784.78	205.47	2.31∼971.34
Winter	29	2.16	1,411.31 ± 1,155.65	432.59	3.33∼943.09

**Notes.**

*n* number of GPS points

PD, patch density, *N* is the number of patches and A is the total area of habitats.

DPL daily path lengths

* *P* < 0.05 of one-way ANOVA test.

## Discussion

### Reasons for limited ranging behavior

A detailed understanding of primate movement ecology offers important information for developing effective policies of wildlife management and conservation (Allen & Singh, 2016). Ren et al. (2009b) studied the correlation between the the DPL of *R. bieti* and ambient temperature. They argued that the reduced DPL in winter was a response to thermoregulatory factors associated with reducing energy expenditure during cold temperatures. Similarly, Liu, Ding & Grueter (2004) suggested that the limited movements of *R. bieti* at Mt. Fuhe represented a compromise between reduced food availability and cold temperatures. In this study, we found that the cost distance for *R. bieti* was significantly greater in the winter than during other seasons of the year, and that DPL also was the shortest in winter, averaging 1,411 m. This is consistent with the contention that several ecological factors including landscape heterogeneity, which was greater in areas exploited during the winter, limited the ranging behavior of *R. bieti* (Deng et al., 2014). In addition, in winter, *R. bieti* occupied the smallest home range (Ren et al., 2009a), supporting the conclusion that increased cost distance and access to large and concentrated food patches affect the ranging behavior of this primate species. We note, however, that given our limited sample size (21 complete days), these conclusions must be viewed as tentative.

### Impact of habitat fragmentation on movement of *R. bieti*

Our study suggests that habitat fragmentation also affected the movement of *R. bieti*. Specifically, they exploited the largest home range and had the greatest DPL in autumn. Autumn represents a time of the year when the mnonkeys utilized less fragmented areas. In contrast during the winter, they exploited highly fragmented habitats had a reduced DPL. This is consistent with a study by Irwin (2008) of the Diademed sifaka. In this lemur species, groups in forest fragments traveled less each day than groups in continuous forest. Habitat disturbance also was reported to have a significant effect on the ranging behavior of roe deer (*Capreolus capreolus*) in southwestern France. These deer were found to avoid disturbed habitats (Coulon et al., 2008).

Fragmented forests are characterized by reduced biodiversity and for some primate species this results in a reduction in food availability and an increase in feeding effort (Virgós, 2001; Zimbres, Peres & Machado, 2017). The strategies of different primates to habitat fragmentation are varied (Onderdonk & Chapman, 2000). In the face of human disturbance, the Sichuan snub-nosed monkey (*Rhinopithecus roxellana*) was found to travel long-distances per day (Li et al., 2005). In areas in which human disturbance was limited, *R. roxellana* traveled approximately 1100 m per day, however, the breeding band traveled 1600 m per day in more fragmented habitats. In contrast, studies have shown that in fragmented forests, primates such as the Diademed sifaka (Irwin, 2008) and the collared brown lemur (*Eulemur collaris*) (Campera et al., 2014) conserve energy and cope with the lower food availability by traveling shorter distance per day. Based on the results of the current study, we found that when exploiting more fragmented habitats in the winter, *R. bieti* was found to exploit a smaller home range and concentrate their ranging activities to lower altitude forests characterized by warmer temperatures. These forest zones contained an abundance of lichen (Grueter et al., 2012; Grueter et al., 2009). Lichens are generally widespread in primary forest and available year-round, making them a key dietary resource for *R. bieti,* who exploits lichen during all months of the year (Grueter et al., 2009; Huang et al., 2017; Xiang et al., 2007).

### The suitability of Yunnan pine forest

Our results suggest that the monkeys alter their pattern of habitat utilization seasonally, likely in response to changes in the distribution and availability of currently available feeding sites. In winter, they spent inhabited Yunnan pine forest (needs more data to support). Yunnan pine forest contains several species of lichens, which can account for more than 80% of winter feeding time (from November to March) (Grueter et al., 2009). Yunnan pine forest has a lower lichen index (1.39) than does spruce-fir forest (which has a lichen index of 1.81), however, the former is found at an elevation of 2,500–3,200 m, which is lower and warmer than spruce-fir forest (3,500–4,000 m) (Grueter et al., 2009; Li et al., 2008). In addition, Yunnan pine forest is phototropic, tends to grow in warmer areas, and therefore may offer thermoregulatoy benefits during the winter (Chen et al., 2012).

In our study we considered Yunnan pine forest to be an unsustainable habitat for *R. bieti* because it contains Matsutake mushrooms, which have a high economic value to members of the local human communities and therefore is characterized by high human disturbance (Li et al., 2008; Xiao et al., 2003). However, Matsutake mushrooms are collected only during the summer and the monkeys were observed to exploit this habitat during the winter. Thus, it is critical to obtain data on seasonal patterns of habitat use for both the human and nonhuman primate communities in order to develop effective conservation solutions for a given species. Although in general, the snub-nosed monkeys avoided areas of high-intensity human disturbance, during the winter most of their activity time occurred in Yunnan pine forest. We hypothesize that due to its warmer microclimate, abundance of lichen, and decreased human activity, Yunnan pine forest represents a critical winter habitat for *R. bieti.* This hypothesis will be tested more rigorously in our future research.

### Reduced DPL in winter

Our results reveal that the DPL of *R. bieti* in winter (1411 m) was shorter than in spring and autumn. These results are consistent with Ren et al. (2009b) who studied this Yunnan snub-nosed monkey breeding band 10 years earlier. In that study, DPL was shortest in the winter (874 m). Similarly, Grueter et al. (2013) studying the same breeding band found that DPL in the winter (985 m) was lower than during other seasons of the year. Our estimate of DPL, was approximately 30% larger than found in previous studies. This could reflect that fact Grueter et al.’s (2013) data were based on a four-season model, and data were collected for one month per season (January, April, July and September). In the case of Ren et al.’s (2009a) and Ren et al.’s (2009b) study, they divided the year into three seasons. In that study, data on breeding band location were collected using a single GPS collar that was affixed to a healthy adult male who was a leader male of a one-male unit (OMU) in the breeding band. Given that OMU females are pregnant during the winter, it is possible that a decrease in winter ranging behavior represents an attempt to reduce prenatal reproductive costs to breeding females. Finally, in our study we collected data for only three month in winter (November, March and April). However, our method of collecting GPS data by tracking the breeding band (similar to the method used by Grueter et al., 2013) was limited because we could only obtain 3-4 location points per day. Therefore, the actual distance traveled per day by wild *R. bieti* remains unclear. However, what was consistent among these three studies was that the monkeys traveled shorter distances per day in the winter than during other times of the year, which could reflect seasonal differences in day length, seasonal differences in resource distribution and availability, or seasonal differences in predation-risk and reproduction.

### Implications for conservation and management

Long-term monitoring of *R. bieti* in both more and less fragmented landscapes contributes to our understanding of patterns of habitat utilization and feeding ecology (Irwin, 2008). Such data are essential for developing management plans for species conservation and habitat restoration. Recent genetic evidence indicates that human-induced habitat fragmentation has led to a decrease in the population size and genetic variability of *R. bieti* (Li et al., 2014). Based on our results, we recommend that improved habitat suitability for the Yunnan snub-nosed monkey requires protecting existing spruce-fir forests, regenerating new spruce-fir forests, constructing corridor between spruce-fir forests and Yunnan Pine Forest in order to promote movement, dispersal, and increased gene flow across breeding bands. These conservation actions represent important steps in protecting and restoring suitable habitat and in decreasing the extinction risk of isolated subpopulations of *R. bieti*.

## Conclusions

Although the strength of our conclusions are limited by small sample size and should be considered preliminary, our results suggest that both the availability of suitable travel routes and habitat fragmentation constrain the movement of *R. bieti*. It appears that snub-nosed monkeys avoid fragmented habitats driven by high-intensity human disturbance, and instead exploit parts of their range characterized by minimal human disturbance and increased food availability. These undisturbed or less disturbed areas contain a high density of lichen, a nutritious and abundant food source for Yunnan snub-nosed monkeys. We recommend that conservation biologists construct detailed maps of landscape heterogeneity, particularly habitat connectivity, forest fragmentation, and seasonal variation in the location of major food patches in order to better understand the effects of anthropogenically influenced patterns of habitat utilization on animal distribution and population viability.

##  Supplemental Information

10.7717/peerj.9165/supp-1 Supplemental Information 1 The DPL and Cost Distance

## Additional Information and Declarations

### Field Study Permissions

The following information was supplied relating to field study approvals (i.e., approving body and any reference numbers): This field experiments were approved by State Forestry Administration of China (the Second National Survey on Terrestrial Wildlife Resources in China).

### Data Availability

The following information was supplied regarding data availability: The raw measurements are available as Supplemental File.
